# Antipsychotic treatment effects and structural MRI brain changes in schizophrenia

**DOI:** 10.1017/S0033291721003809

**Published:** 2023-04

**Authors:** Robin Emsley, Stefan du Plessis, Lebogang Phahladira, Hilmar K. Luckhoff, Frederika Scheffler, Sanja Kilian, Retha Smit, Chanelle Buckle, Bonginkosi Chiliza, Laila Asmal

**Affiliations:** 1 Department of Psychiatry, Stellenbosch University, Tygerberg Campus, Cape Town, South Africa; 2 Department of Psychiatry, Nelson R Mandela School of Medicine, University of Kwazulu-Natal, Durban, South Africa

**Keywords:** Antipsychotic, brain structure, MRI, schizophrenia, white matter, basal ganglia, cortical thickness

## Abstract

**Background:**

Progressive brain structural MRI changes are described in schizophrenia and have been ascribed to both illness progression and antipsychotic treatment. We investigated treatment effects, in terms of total cumulative antipsychotic dose, efficacy and tolerability, on brain structural changes over the first 24 months of treatment in schizophrenia.

**Methods:**

A prospective, 24-month, single-site cohort study in 99 minimally treated patients with first-episode schizophrenia, schizophreniform and schizoaffective disorder, and 98 matched healthy controls. We treated the patients according to a fixed protocol with flupenthixol decanoate, a long-acting injectable antipsychotic. We assessed psychopathology, cognition, extrapyramidal symptoms and BMI, and acquired MRI scans at months 0, 12 and 24. We selected global cortical thickness, white matter volume and basal ganglia volume as the regions of interest.

**Results:**

The only significant group × time interaction was for basal ganglia volumes. However, patients, but not controls, displayed cortical thickness reductions and increases in white matter and basal ganglia volumes. Cortical thickness reductions were unrelated to treatment. White matter volume increases were associated with lower cumulative antipsychotic dose, greater improvements in psychopathology and cognition, and more extrapyramidal symptoms. Basal ganglia volume increases were associated with greater improvements in psychopathology, greater increases in BMI and more extrapyramidal symptoms.

**Conclusions:**

We provide evidence for plasticity in white matter and basal ganglia associated with antipsychotic treatment in schizophrenia, most likely linked to the dopamine blocking actions of these agents. Cortical changes may be more closely related to the neurodevelopmental, non-dopaminergic aspects of the illness.

## Introduction

Antipsychotics have been the mainstay of treatment for schizophrenia since the 1950s, and their beneficial effects are well documented (Leucht et al., 2012). Earlier studies suggested that antipsychotics protect against the ‘toxic’ effects of unmitigated illness (Wyatt, 1991), although more recently, attention has shifted to a possible ‘neurotoxic’ effect of antipsychotics. Greater antipsychotic exposure has been linked to poorer outcome (Wunderink, Nieboer, Wiersma, Sytema, & Nienhuis, 2013) and brain volume reductions (Ho, Andreasen, Ziebell, Pierson, & Magnotta, 2011) leading some to question the need for the routine long-term use of antipsychotic medication (Murray et al., 2016).

Differences in brain structure have been extensively described in schizophrenia compared to healthy controls, including widespread, albeit subtle, reductions in cortical grey matter (van Erp et al., 2018) and both reductions and increases in subcortical (van Erp et al., 2016) and white matter volumes (Makris et al., 2010). While some differences are evident prior to (Cannon et al., 2015), and at the onset of, first psychotic symptoms (Vita, De, Silenzi, & Dieci, 2006), further progressive changes have been described, especially in the early years of illness (Olabi et al., 2011). The role of antipsychotic medication in either mitigating or contributing to these changes is not clear. On the one hand, a relationship between brain volume reductions and poorer treatment outcomes suggests a neurodegenerative component to the illness, and that antipsychotics are ‘neuroprotective’ (Lieberman et al., 2005). On the other hand, considerable evidence suggests that antipsychotics themselves cause brain volume changes. A study in primates reported brain volume reductions with therapeutic doses of both haloperidol and olanzapine (Dorph-Petersen et al., 2005). Also, longitudinal studies in patients with schizophrenia have reported brain volume reductions that were associated with the estimated exposure to antipsychotic medication (Guo et al., 2015; Ho et al., 2011). First- and second-generation antipsychotics have been reported to affect brain volumes differentially, with larger global reductions and greater basal ganglia increases being associated with first generation antipsychotics (van Haaren, Cahn, Hulshoff Pol, & Kahn, 2013). Finally, systematic reviews of retrospective data report an association between antipsychotic exposure and grey and white matter volume reductions (Haijma et al., 2013; Huhtaniska et al., 2017; Vita, De, Deste, Barlati, & Sacchetti, 2015), and basal ganglia volume increases (Navari & Dazzan, 2009), although most did not find a linear relationship between the degree of antipsychotic exposure and progressive brain changes (Roiz-Santianez, Suarez-Pinilla, & Crespo-Facorro, 2015).

Interpretation of the results of studies to date is made difficult by several methodological considerations (Guo et al., 2015). These studies seldom focused primarily on the relationship between antipsychotic medication and brain volume changes, and most were either cross-sectional or naturalistic. Treatment was mostly not standardised, with antipsychotic exposure being estimated retrospectively and adherence not objectively assessed. Furthermore, imaging methodology varied across studies, often with non-uniform time-points, multiple scan sites with different protocols, and different brain regions selected.

In this study, designed specifically to examine brain structural MRI changes in relation to antipsychotic treatment, we addressed several of the above methodological issues. Our goal was to characterise the contributions of different aspects of antipsychotic treatment to structural MRI brain changes in schizophrenia. We investigated treatment effects in terms of cumulative antipsychotic dose, efficacy (changes in psychopathology and cognition) and adverse effects (weight gain and extrapyramidal symptoms). We hypothesised that, compared with healthy controls, the patients would experience reductions in cortical thickness and white matter volumes and increases in basal ganglia volumes, and that these changes would be differentially associated with antipsychotic dose, efficacy and adverse effects.

## Methods

### Study design and ethical approval

This was a prospective, longitudinal, single-site cohort study, conducted between 2011 and 2017. We obtained ethical approval from the Health Research Ethics Committee at the Faculty of Medicine and Health Sciences, Stellenbosch University (N06/08/148). Patients and/or their legal guardians provided written, informed consent.

### Participants

We recruited in- and out-patients from first contacts at psychiatric hospitals and community clinics within a well-defined catchment area in Cape Town and surrounding districts. Inclusion criteria were age 16–45 years, diagnosis of schizophrenia, schizoaffective or schizophreniform disorder according to the Diagnostic and Statistical Manual of Mental Diseases, Fourth Edition (DSM-IV). Exclusion criteria were a lifetime exposure to antipsychotics of more than 4 weeks; previous treatment with a long-acting injectable antipsychotic; a serious or unstable medical condition; educational level <grade 7; or current diagnosis of substance abuse or dependence, or substance induced psychotic disorder (DSM-IV). Healthy controls were from the same catchment area, with similar socioeconomic circumstances to the patients. They were recruited by neighbourhood contacts of the families of the patients, as well as from advertisements placed in community centres. Controls were matched for age, sex and ethnicity. They were excluded if they had a first-degree relative with a psychotic disorder or if they had a DSM-IV axis I or II disorder. Patients and controls were assessed with the Structured Clinical Interview for DSM-IV (SCID) (First, Spitzer, & Williams, 1994). Participants were compensated for transport costs incurred during their participation in the study but did not receive any other financial reward.

The study was conducted in a research unit based in an academic psychiatric hospital. Patients were seen at 2-weekly intervals by the study nurses throughout the study, for administration of the study medication. Psychoeducation was provided to all patients and carers. Family therapy and substance-use interventions were offered where appropriate. Clinical assessments were conducted at five time-points during the initial 3 months, and every 3 months after that. Cognitive assessments were conducted at 6 monthly intervals, and MRI scans were obtained at 0, 12 and 24 months.

### Clinical and cognitive assessments

Psychopathology was assessed by physicians using the Positive and Negative Syndrome Scale (PANSS) (Kay, Fiszbein, & Opler, 1987), and training and inter-rater reliability testing was periodically conducted (intra-class correlation 0.7 or higher). We calculated PANSS factor analysis-derived positive, negative and disorganised domain scores as previously described (Emsley et al., 2003). The Extrapyramidal Symptom Rating Scale (ESRS) (Chouinard & Margolese, 2005) was used to measure treatment-related movement disorders. Cognitive performance was assessed by the MATRICS Cognitive Consensus Battery (MCCB), administered by trained psychologists. Age and sex-corrected norms were used according to the manual guidelines, and we used the MCCB Composite score as our measure of global cognition (Nuechterlein & Green, 2006). We determined cannabis use by patient and carer report, together with repeated urine toxicology testing at months 0, 3, 6, 12, 18 and 24. To quantify the degree of persistent cannabis use, we summed the number of positive urine tests, as a discrete variable. For body mass measurements, patients removed all surplus clothing and were weighed on an electronic scale that was regularly calibrated. Height was measured with a prefixed, wall-mounted measuring tape.

### Study treatment

Patients were treated according to a fixed protocol with flupenthixol decanoate, a long-acting injectable antipsychotic. Flupenthixol antagonises dopamine at D1, D2, D3 receptors, as well as 5-HT2A and 5-HT2C and *α*1-adrenergic receptors. Its pharmacological profile has similarities to several newer generation antipsychotics (Mahapatra, Quraishi, David, Sampson, & Adams, 2014). There was a week lead-in with oral flupenthixol 1–3 mg/day followed by flupenthixol decanoate intramuscular injections 2-weekly for the study duration. Initiation dose was 10 mg 2-weekly, with 6-weekly increments of 10 mg if necessary, to a maximum of 30 mg 2-weekly. We followed a low-dosing strategy, initiating antipsychotic treatment at the lowest possible dose, treating initial agitation with a benzodiazepine rather than increasing the antipsychotic dose, and gradual upward titration of the antipsychotic dose only when necessary, until optimal response was obtained. Permitted concomitant medications included lorazepam, anticholinergics, propranolol, antidepressants and medications for general medical conditions. No benzodiazepines, propranolol or anticholinergics were allowed in the 12 h prior to the clinical, cognitive and MRI assessments. Prohibited medications included other antipsychotics, mood stabilisers and psychostimulants. Six participants were treated with long-acting risperidone injection for the first 12 weeks of the study, before being switched to flupenthixol decanoate. For these patients, there was a week lead-in of oral risperidone, continued for 3 weeks. The starting dose for long-acting risperidone was 25 mg IMI 2-weekly.

We were able to calculate the total antipsychotic load received by each patient with precision. We recorded the date and dose of each injection and each oral tablet prescribed. Doses were converted to oral flupenthixol milligram equivalents, according to consensus-derived guidelines for dose equivalencies (Gardner, Murphy, O'Donnell, Centorrino, & Baldessarini, 2010), and summed to provide the total cumulative antipsychotic dose.

### Imaging methods

Patients underwent baseline scans before receiving any study antipsychotic medication. We acquired high-resolution T1-weighted data on a research-dedicated 3 T Siemens Allegra MRI scanner (Erlangen, Germany) with the following acquisition parameters: MPRAGE sequence, 2080 ms repetition time, 4.88 ms echo-time, field of view: 230 mm, 176 slices, 0.9 mm × 0.9mm × 1 mm voxel size. We screened all scans for intracranial pathology and motion artefacts. Scans were processed using FreeSurfer version 6 (http://surfer.nmr.mgh.harvard.edu/). Slices were resampled to a three-dimensional image with 1 mm isotropic voxels. Non-uniform intensity normalisation was performed and images registered to the Montreal Neurological Institute space. A second normalisation step was performed with control points automatically identified and normalised to a standard intensity value, followed by an automated skull strip procedure. Gross brain anatomy was delineated into cortical and subcortical labels. Reconstructions were performed with custom batching scripts, on the Centre for High Performance Computing, Cape Town, Sun Intel Lengau cluster (http://www.chpc.ac.za/). All of the data were visually inspected for errors in Talairach transformation, skull strip, final segmentations and within-subject registrations. Errors were corrected manually and re-inspected. Detailed quality assessment was conducted according to the ENIGMA consortium QC protocol (www.enigma.ini.usc.edu). Scans that did not meet the threshold for reasonable quality or could not be processed successfully were excluded from all analyses.

### Measures of brain morphology

We selected, *a priori*, three brain regions that we considered most important in relation to treatment: (1) *Global cortical thickness:* We averaged the cortical thickness measures for the left and right hemispheres and used the surface area of each hemisphere as a weighting factor, as recommended (https://surfer.nmr.mgh.harvard.edu/fswiki/UserContributions/FAQ). We selected a global measure rather than specific cortical regions, as antipsychotic dose was reported to affect grey matter globally rather than selectively (Torres et al., 2016). (2) *White matter volume:* This was the sum of the left and right cerebral white matter volume measures. (3) *Basal ganglia volume:* We selected the caudate, putamen and pallidum (Chand et al., 2020; Ebdrup, Norbak, Borgwardt, & Glenthoj, 2013; Okada et al., 2016; van Erp et al., 2016), creating a single measure by summing their left and right volumes. White matter and basal ganglia volumetric measures were corrected for intracranial volumes and expressed as a percentage of the estimated total intracranial volume (%eTIV), and cortical thickness expressed as mm (https://surfer.nmr.mgh.harvard.edu/fswiki/eTIV).

### Data analysis

Our analyses were conducted on the intent to treat population comprising all the entered participants with baseline clinical data and at least one MRI scan. We conducted statistical analyses with Statistica version 13.0 (Dell, 2015). Differences in demographic and clinical characteristics between patients and controls were compared by two-sample *t* tests and χ^2^ tests for continuous and categorical variables, respectively. All tests were two-tailed. We used mixed model repeated measures analysis of variance (MMRM) in two sets of *a priori* analyses, with restricted maximum likelihood estimation for fitting the linear mixed models. (1) In the first set, we compared the brain changes over time in the patients *v.* controls. We entered the brain regions separately, as dependent variables, modelled as repeated measures. As a random effect, we specified intercepts for participants. Time was a grouping variable, group × time interaction was a fixed effect. Level of education was the only demographic variable to differ between patients and controls and therefore was entered as a time invariant covariate. (2) In the second set of analyses, we sought relationships between brain changes and treatment effects in the patient group only. Total cumulative antipsychotic dose was entered into our model as a time-invariant fixed effect. PANSS total score and BMI were time-dependent predictors. Extrapyramidal symptoms were generally mild and transient, and ESRS Total scores did not change significantly over the course of treatment (*p* = 0.1431). We therefore calculated an ESRS Total change to maximum score, as the change from baseline to the highest score attained at any time-point. This was entered as a time-invariant fixed effect. Covariates were age, gender, level of education, number of cannabis-positive tests and the baseline value for the dependent variable. We assessed the cognitive effects on brain morphology in a separate analysis, as cognitive data were not available for all the participants (see below). The MCCB Composite score was added as a time-dependent fixed effect to the above model. For all of the MMRM analyses we used Fisher's Least Significant Difference (LSD) test for within-analysis post-hoc comparisons and applied Benjamini and Hochberg false discovery rate (FDR) corrections with a *q* value of 0.05 for multiple comparisons (Genovese, Lazar, & Nichols, 2002). We established directionality of the significant fixed effects by partial correlational analyses.

Finally, to test the robustness of our findings, we conducted two sets of sensitivity analyses. The first was a completer analysis, including only the participants who completed 2 years follow-up and had a M24 MRI scan. In the second, we re-ran the MMRM for white matter volume using raw values uncorrected for eTIV, as the primary analysis delivered unanticipated findings.

## Results

Of 126 patients entered, 99 had baseline data and at least one MRI scan and were included in the analysis. Reasons for exclusion were scanner unavailability (*n* = 20), poor scan quality (*n* = 2), scans lost on the server (*n* = 2), patient refused scan (*n* = 2) and protocol violation (*n* = 1). Of the 99 patients included, 53 completed the 24 months of treatment. Reasons for dropout were absconded (*n* = 19), consent withdrawal (*n* = 9), lack of treatment efficacy (*n* = 8), relocated (*n* = 4), no longer met inclusion criteria (*n* = 2), substance abuse (*n* = 1), incarceration (*n* = 1), severe side effects (*n* = 1) and death (*n* = 1). The treatment response was generally favourable, with 79 (80%) achieving operationally defined remission criteria (Andreasen et al., 2005) at endpoint. The control group comprised 98 matched, healthy volunteers. The number of suitable scans at each time-point for patients and controls respectively, was M0: 95 and 97; M12: 45 and 58; M24: 48 and 35. (For the MCCB cognitive data, the numbers for patients and controls respectively, were M0: 76 and 95; M12: 46 and 59; M24 39 and 40.) We provide the baseline demographic, cognitive and MRI characteristics for the patients and controls in Table 1. The patients had lower educational levels and MCCB Composite scores (*p* = 0.0003) and thinner global cortical thickness (*p* = 0.0335). Table 1 also provides baseline clinical and treatment characteristics for the patients.

**Table 1.** Baseline demographic, cognitive and brain MRI characteristics of the patients and healthy controls, and baseline clinical and treatment characteristics for the patients

	Patients (*N* = 99)		Controls (*N* = 98)			
Characteristic	*N*	%	*N*	%	*t*	*p*
Sex, male	74	75	61	62	3.57	0.0589
Ethnicity					0.001	0.9993
Mixed	77	78	76	78		
Black	15	15	15	15		
White	7	7	7	7		
Cannabis test positive	34	34				
DSM-IV diagnosis						
Schizophrenia	69	70				
Schizophreniform	29	29				
Schizoaffective	1	1				
	Mean	s.d.	Mean	s.d.	χ^2^	*p*
Age (years)	24.35	6.66	25.94	7.38	−1.59	0.1138
Highest school grade passed	9.89	2.09	10.47	1.51	−2.21	0.0281
Global cortical thickness (mm)	2.4233	0.1322	2.4662	0.1443	−2.14	0.0335
White matter volume (%eTIV)	319 515	3.0811	31.8330	3.9587	0.23	0.8177
Basal ganglia (%eTIV)	1.3861	0.1373	1.3891	0.1562	−0.14	0.8882
DUP (weeks)	35,98	45.81				
Time in the study (weeks)	66,73	40.33				
Modal flupenthixol decanoate dose (mg 2-weekly)	11,92	3.89				
% Adherence	98,62	3.48				
Cumulative antipsychotic dose (flupenthixol mg equiv.)	146 617	985.69				
PANSS Total score	93,43	15.15				
PANSS Positive factor	17,53	3.30				
PANSS Negative factor	19,40	5.43				
PANSS Disorganised factor	11,89	3.08				
MCCB Composite score	21.68	13.23				
ESRS Total score baseline	1,03	2.78				
ESRS Total score change baseline to maximum score	3,38	5.18				
BMI (kg/m)	21,79	3.98				

DUP, duration of untreated psychosis; PANSS, Positive and Negative Syndrome Scale; MCCB, MATRICS Consensus Cognitive Battery; ESRS, Extrapyramidal Symptom Rating Scale; BMI, body mass index.

### Brain MRI changes for the patients *v*. healthy controls

**Fig. 1.**
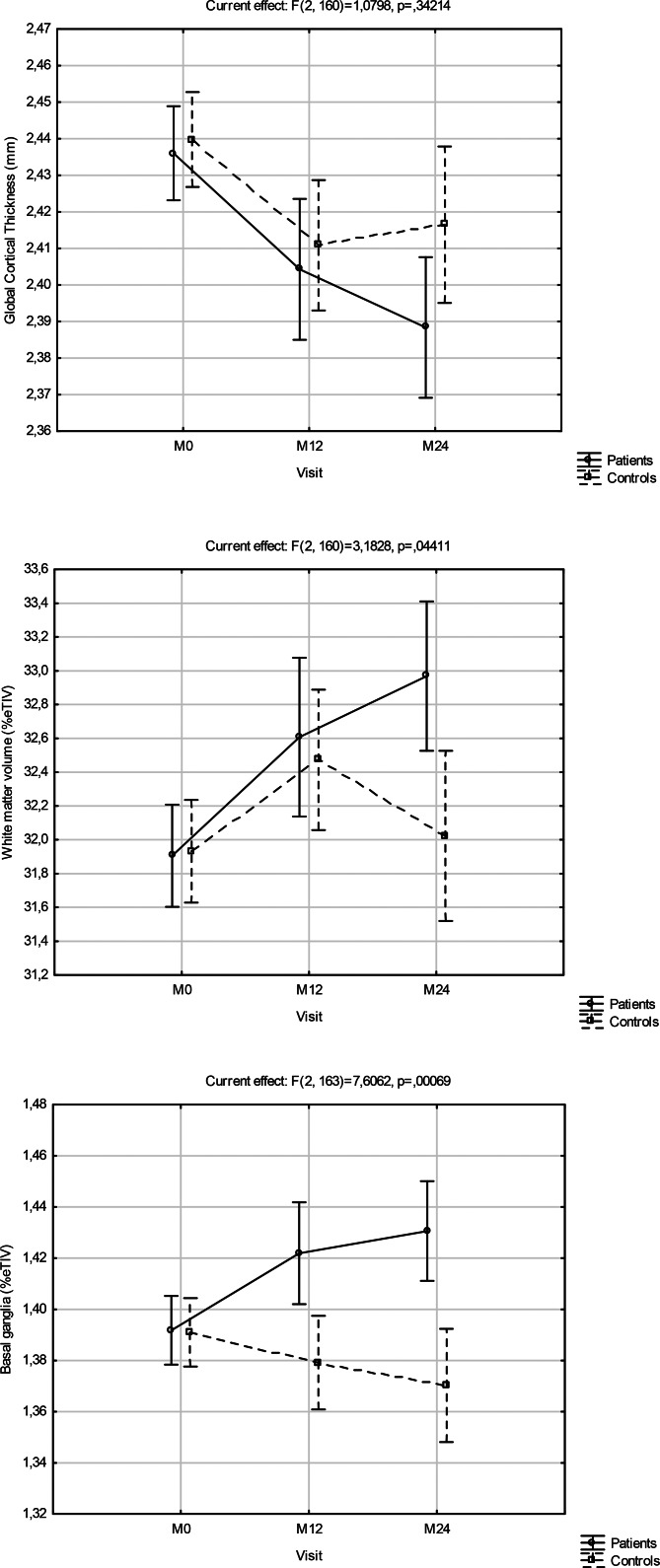
Brain structural MRI changes for the patients *v*. controls, as visit-wise least square means and 95% confidence intervals from baseline to month 24, from the MMRM models.

Figure 1 shows the brain structural MRI changes for the patients *v.* controls, as visit-wise least square means and 95% confidence intervals (95% CI) from baseline to month 24, from the MMRM models. Table 2 details the changes (LSD means and 95% CI) for the three MRI brain regions from baseline to M24 for patients and controls and the fixed effects of group, time and group × time interaction, adjusted for level of education and baseline MRI value. After FDR correction (significance level 0.0241), basal ganglia volume was the only brain region that showed a significant group × time interaction effect (*p* = 0.0007). Post hoc testing (LSD corrected) indicated significant reductions in the patients from baseline to M24 for global cortical thickness (*p* = 0.0001) and increases for white matter volume (*p* = 0.0001) and basal ganglia volume (*p* = 0.0007), and no significant changes in the controls.

**Table 2.** Changes from baseline to M24 for patients and controls, and fixed effects of group, time and group × time interaction for the four MRI brain regions, adjusted for level of education and baseline MRI value

	Change from baseline to M24^a^				Fixed effect test		
	Mean	95% CI	*p* Value			*F*	*p* Value
Global cortical thickness (mm)							
Patients	−0.0477	−0.0703 to −0.0250	0.0001		Group	1.88	0.1727
Controls	−0.0233	−0.0478 to 0.0012	0.0626		Time	12.88	0.0001
					Group × time	1.08	0.3421
White matter volume (%eTIV)							
Patients	1.0627	0.5471 to 1.5783	0.0001		Group	1.67	0.1982
Controls	0.0913	−0.4779 to 0.6604	0.7519		Time	8.43	0.0003
					Group × time	3.18	0.0441
Basal ganglia volume (%eTIV)							
Patients	0.0388	0.0165 to 0.0610	0.0007		Group	9.92	0.0019
Controls	−0.0207	−0.0453 to 0.0039	0.0979		Time	1.15	0.3198
					Group × time	7.61	0.0007

CI, confidence interval; %eTIV, percentage of estimated total intracranial volume.

a Fisher's least significant difference test.

### Antipsychotic treatment effects on brain MRI changes

Table 3 provides the results of the MMRM for the treatment-related effects on brain MRI changes. After FDR correction, higher cumulative antipsychotic dose predicted lesser increase in white matter volume (*p* = 0.0120); greater reduction in PANSS Total scores predicted larger increases in white matter (*p* = 0.0194) and basal ganglia (*p* = 0.0187) volumes; greater improvements in MCCB Composite scores predicted greater increases in white matter volume (*F* = 8.72, *p* = 0.006); greater increase in BMI predicted greater increase in basal ganglia volume (*p* = 0.0001); and greater baseline to maximum ESRS Total scores predicted greater increase in white matter (*p* = 0.0012) and basal ganglia volumes (*p* = 0.0001). Partial correlations controlling for age, sex and education indicated that the cumulative antipsychotic dose was significantly negatively correlated with the PANSS Total score (*r* = −0.2540, *p* < 0.0001), and significantly positively correlated with BMI (*r* = 0.1371, *p* = 0.03), but not with ESRS Total scores (*r* = 0.0325, *p* = 0.595) or MCCB Composite scores (*r* = −0.0498, *p* = 0.529).

**Table 3.** Fixed effects for cumulative antipsychotic dose, PANSS Total score, BMI and ESRS Total change to maximum score on the brain MRI regions

	Global cortical thickness		White matter volume			Basal ganglia		
Fixed effect	*F*	*p*	*F*	*p*	Direction^a^	*F*	*p*	Direction^a^
Total cumulative antipsychotic dose	1.23	0.2705	6.66	0.0120	−	0.86	0.3566	
PANSS Total score	3.58	0.0627	5.73	0.0194	−	5.79	0.0187	−
MCCB Composite score	0.71	0.4065	8.72	0.006	+	1.01	0.3225	
BMI	0.04	0.8437	3.16	0.0799		20.92	<0.0001	+
ESRS Total change score	1.04	0.3118	11.33	0.0012	+	30.77	<0.0001	+

PANSS, Positive and Negative Syndrome Scale; MCCB, MATRICS Cognitive Consensus Battery; BMI, body mass index; ESRS, Extrapyramidal Symptom Rating Scale.

a Direction of significant associations, determined by partial correlations.

### Sensitivity analyses

Results of the first set of sensitivity analyses including completers only were largely similar to those of the primary analyses. The group × time interaction effects for cortical thickness changes (*F* = 1.0856, *p* = 0.3408) and white matter volume changes (*F* = 2.4787, *p* = 0.0878) were not significant, while for basal ganglia volume changes they were (*F* = 4.0895, *p* = 0.01893). Post hoc LSD testing indicated significant reductions in the patients from baseline to M24 for global cortical thickness (*p* = 0.0006), increases for white matter volume (*p* = 0.001) and basal ganglia volume (*p* = 0.0182), and no significant changes in the controls. For the treatment-related fixed effects on the brain regions in the patients, most of the results were similar to the primary analyses. Higher cumulative antipsychotic dose predicted less increase in white matter volume (although only at the uncorrected significance level) (*F* = 0.265; *p* = 0.0265); greater reduction in PANSS Total scores predicted, at uncorrected significance levels, larger increases in white matter (*F* = 0.63, *p* = 0.0352) and basal ganglia (*F* = 4.00, *p* = 0.0498) volumes; increases in BMI predicted greater increase in basal ganglia volume (*F* = 12.35, *p* = 0.0008), and greater baseline to maximum ESRS Total change scores predicted greater increase in white matter (*F* = 12.88, *p* = 0.0007) and basal ganglia volumes (*F* = 37.29, *p* = 0.0001). The only substantial differences from the primary analyses were that greater reduction in PANSS Total scores significantly predicted less reduction in global cortical thickness (*F* = 9.15; *p* = 0.0036) and MCCB Composite scores no longer predicted white matter volume changes (*F* = 00.26, *p* = 0.6117).

For the second sensitivity analysis using raw values for white matter volumes, results again followed a similar pattern to those of the primary analysis. The group × time effect was only significant at the uncorrected level (*F* = 3.33, *p* = 0.0383) and LSD testing indicated significant increases from baseline to M24 for patients (*p* < 0.0001) but not controls (*p* = 0.8500). In the patients only there were significant main effects for PANSS Total (*F* = 16.18, *p* = 0001) and ESRS Total change scores (*F* = 26.95, *p* < 0.0001), but not for MCCB Composite score (*F* = 2.93, *p* = 0.0901), BMI (*F* = 2.97, *p* = 0.0892) or cumulative antipsychotic dose (*F* = 1.94, *p* = 0.1685).

### Cannabis use and brain MRI changes

We entered the number of positive cannabis urine tests over the study duration as a covariate into the MMRM models investigating the treatment effects. More frequent positive tests were associated with lesser reductions in cortical thickness (*F* = 8.92, *p* = 0.0039) and lesser increases in white matter volumes (*F* = 18.65, *p* = 0.0001), but not with basal ganglia volume changes (*F* = 0.72, *p* = 0.3975).

### Secondary analyses

To investigate whether the significant effects that we found for PANSS Total scores and MCCB Composite scores were domain-specific, we conducted secondary analyses with the MMRM models constructed as in the main analyses, but in this case with the PANSS domain scores and the MCCB domain scores entered as time-dependent fixed effects. In these analyses, we did not correct for multiple comparisons and the findings are exploratory only. For the PANSS domains, we found a significant effect for positive symptoms for both white matter volume and basal ganglia volume changes, respectively (*F* = 12.02, *p* = 0.0009 and *F* = 8.74, *p* = 0.004), but not for negative (*F* = 0.51, *p* = 0.4774 and *F* = 0.001, *p* = 0.9752) or disorganised (*F* = 1.27, *p* = 0.2634 and *F* = 0.25, *p* = 0.6194) symptoms. We found no significant effects for any of the MCCB domains on white matter volume (*p* > 0.05). Finally, we assessed the effects of hospitalisation or relapse during the study on brain structural changes. There were no significant associations for hospitalisation, duration of hospitalisation or relapse, respectively, on cortical thickness (*F* = 0.46, *p* = 0.4907; *F* = 0.13, *p* = 0.7241; *F* = 0.00, *p* = 0.9969) white matter volume (*F* = 0.49, *p* = 0.4866; *F* = 0.43, *p* = 0.5170; *F* = 0.03, *p* = 0.8684) or basal ganglia volume (*F* = 3.88, *p* = 0.0525; *F* = 1.54, *p* = 0.2237; *F* = 0.61, *p* = 0.4361) changes, respectively.

## Discussion

In this study, we found reductions in global cortical thickness and increases in white matter and basal ganglia volumes over time in patients, but not in controls, although basal ganglia volumes were the only region to show a significant group × time interaction. We also found differential treatment effects in the three brain regions in the patients.

### Global cortical thickness

While the group × time interaction did not differ significantly between individuals with schizophrenia and healthy controls, we found several significant differences in global cortical thickness in the post-hoc tests. At baseline, patients had thinner global cortical thickness measures, and further small, but significant, reductions occurred over 24 months in patients, but not in controls. These findings are consistent with a meta-analysis of longitudinal studies reporting progressive loss in cortical grey matter volume, with the most significant reductions occurring in the early stages of the disease (Vita, De, Deste, & Sacchetti, 2012). The literature is mixed however, with some reporting no progressive changes (Haukvik et al., 2016), and even a reversal of baseline deficits (Schaufelberger et al., 2011).

The reductions in global cortical thickness were independent of treatment effects, insofar as they were not associated with cumulative antipsychotic dose, efficacy or adverse effects. This differs from the findings of the ENIGMA consortium meta-analysis reporting widespread cortical thinning in schizophrenia that was associated with higher medication dose, higher positive symptom scores in some regions and higher negative symptom scores in other regions (van Erp et al., 2018). (In this regard, it should be noted that we did find an association between greater reductions in PANSS Total scores and lesser reductions in cortical thickness in the completers analysis.) Our failure to find an association between cortical thickness changes and treatment effects in our primary analysis also differs from the findings of a meta-analysis of longitudinal studies that showed greater grey matter volume reductions in patients that were related to cumulative antipsychotic intake, but not to symptom severity (Vita et al., 2015). While the differences between our findings and the above meta-analyses could be due to the greater statistical power of those studies, they could also be due to the distinct characteristics of our sample and study methodology. Thus, results of studies conducted in chronic samples and in naturalistic settings could be confounded by factors such as treatment non-adherence, previous treatment and illness chronicity. Our findings suggest that, in the early years of treatment of schizophrenia, in patients with assured treatment and a favourable response, subtle reductions in cortical thickness occur that are not related to the degree of exposure, efficacy or lack thereof, or adverse effects of antipsychotic treatment. Our results are also consistent with a recent study using machine learning on MRI data that identified two distinct neuroanatomical subtypes in schizophrenia. The first, including widespread grey matter volume reductions, is proposed to be related to the non-dopaminergic neurodevelopmental abnormalities in schizophrenia, and less responsive to dopamine-blocking antipsychotics (Chand et al., 2020).

### White matter volumes

While the group × time interaction effect did not meet our adjusted significance level, we again found significant differences between individuals with schizophrenia and healthy controls in the post-hoc tests. The increase that we observed in white matter volumes over the course of treatment was unanticipated, given the reports of smaller white matter volumes in both unmedicated and medicated patients with schizophrenia (Haijma et al., 2013), and a meta-analysis of longitudinal studies reporting progressive reductions in white matter volumes (Olabi et al., 2011). However, not all longitudinal studies found white matter volume reductions (Lieberman et al., 2005), and a cross-sectional study found both increases and decreases in patients with schizophrenia compared to healthy controls, with larger white matter volumes being associated with positive symptoms and smaller volumes with negative symptoms (Makris et al., 2010). It could be argued that the increases that we observed in our patients could be explained by normal aging, as white matter volume is reported to increase with age until approximately the fifth decade of life in healthy individuals (Bartzokis et al., 2001). However, counting against this is that our healthy controls did not show similar increases over the 2-year study period.

Our finding of improvements in psychopathology and cognition being related to greater increases in white matter volumes suggests a link between antipsychotic efficacy and white matter changes (although the association with cognition was no longer significant in the sensitivity analyses). Such a relationship is further supported by the inverse association that we found in the primary analysis between cumulative antipsychotic dose and white matter volume increase, as higher doses are more likely to be prescribed in patients responding less well to treatment. This provides a possible explanation for the results of a long-term study reporting progressive decrement in white matter volume that was most evident among patients who received more antipsychotic treatment (Ho et al., 2011), and in the same cohort, that greater white matter reductions occurred in patients who spent more time in relapse (Andreasen, Liu, Ziebell, Vora, & Ho, 2013). Thus, with assured antipsychotic treatment via a long-acting injectable formulation, a favourable treatment response appears to be accompanied by increases in white matter volume, while in the longer term, white matter volume reductions may be linked to periods of suboptimal adherence, illness recurrence and emergent refractoriness. Further support for this possibility is forthcoming from reported differential effects for long-acting injectable *v.* oral antipsychotics on white matter volume in schizophrenia. A small randomised, controlled trial conducted over 6 months found that white matter volumes remained stable in patients receiving risperidone long-acting injection (*n* = 11), whereas those treated with oral risperidone (*n* = 13) showed volume reductions. Those authors proposed that antipsychotics stabilise white matter via a promyelination effect, and that long-acting injectable antipsychotics may have an advantage over their oral counterparts in achieving this effect via improved adherence (Bartzokis et al., 2011). A problem with this hypothesis, however, is that our patients did not have smaller white matter volumes at baseline, and volumes increased beyond those of controls during treatment, suggesting excessive white matter increases rather than normalisation.

### Basal ganglia

This study provides compelling evidence for basal ganglia volume increases in schizophrenia that are related to antipsychotic treatment rather than to the underlying illness. The similar basal ganglia volumes in patients and controls at baseline are consistent with previous reports in first episode, including treatment-naïve samples (Brandt & Bonelli, 2008), suggesting no pre-treatment structural MRI differences. The volume increases that we found with treatment are consistent with the reports of basal ganglia enlargement in chronic, treated samples (Ebdrup et al., 2013; van Erp et al., 2016). Longitudinal studies to date have, however, reported inconsistent findings. Earlier studies suggested a differential effect for antipsychotic class, with increased basal ganglia volume being associated with treatment with first-generation antipsychotics, but not second-generation antipsychotics (Navari & Dazzan, 2009). Later systematic reviews reported both increases and decreases in basal ganglia volumes, and not specific to any antipsychotic class (Ebdrup et al., 2013; Huhtaniska et al., 2017).

The increases that we found in basal ganglia volume were linked to both the efficacy and adverse effects of antipsychotic treatment, pointing to a shared underlying mechanistic pathway, and most likely involving dopamine. Dorsal striatal dopamine dysfunction underlies the symptoms of psychosis (McCutcheon, Beck, Jauhar, & Howes, 2018), and the efficacy of antipsychotics is related to their antagonistic effects at the dopamine D2 receptor (Miyamoto, Miyake, Jarskog, Fleischhacker, & Lieberman, 2012). Extrapyramidal symptoms are mediated by blockade of D2 receptors in the nigrostriatal system (Sykes et al., 2017), and antipsychotic-induced weight gain has been linked, at least in part, to its D2 and D3 antagonistic effects (Dayabandara et al., 2017). Therefore, our findings are consistent with the basal ganglia being related to the hyperdopaminergic component of the illness, as proposed by Chand et al. (2020). In their machine learning-derived identification of neuroanatomical subtypes for schizophrenia, Subtype 2 was characterised by increased volume in the basal ganglia, together with some white matter, especially in the internal capsule.

While our study design allowed us to address several methodological shortcomings of previous studies, some limitations need to be considered. First, there is a risk of misinterpreting the meaning of the observed brain changes. MRI data are not a direct measure of brain structure, and are potentially confounded by epiphenomena and artefacts, and differences between patients *v.* controls are not necessarily evidence of structural abnormalities, or potentially deleterious effects of treatment (Weinberger & Radulescu, 2020). Second, the sample size is limited, and may not have had sufficient power to detect small effect sizes. However, for a single-site study, the sample is relatively large, and power limitations are countered by the advantages of homogeneity of the sample and standardised treatment and assessments, including using a single scanner. Third, as with most longitudinal studies in psychosis, participant attrition was considerable, introducing the possibility of measurement error associated with missing values. Although our use of MMRM models offers a powerful approach to dealing with missing values, the assumption of missingness at random when predicting missing values may introduce error. Nevertheless, our completers-only sensitivity analyses produced largely similar results, indicating that our findings were not an artefact of the intent to treat population. Another potential limitation of the high attrition rate that may have biased our findings is that the retained patients may not be representative of the entire sample, particularly as several participants directly, and likely indirectly, withdrew for treatment-related reasons. Subsequently, this study does not permit inferences regarding illness progression, and results can only be considered in the context of patients generally responding favourably to treatment. Fourth, it is entirely plausible that the brain regions we selected do not optimally identify treatment effects on brain structure, and that further regional specificity might apply. We also did not assess possible laterality effects. Fifth, volumetric analysis of brain structures is confounded by inter-individual variability in brain morphology and total head size. In the primary analyses, we corrected our volumetric measures for eTIV, as described by the developers of FreeSurfer (https://surfer.nmr.mgh.harvard.edu/fswiki/eTIV). eTIV is consistent over time and correcting regional volumes by eTIV is considered important to estimate the extent of change from a premorbid state (Voevodskaya et al., 2014). In any event, our similar findings with the uncorrected raw white matter volumes further support the validity of the results of the primary analysis.

Sixth, the study duration of 2 years does not address the longer-term effects of antipsychotics on brain structure. Finally, while the use of a single antipsychotic removed the effects of treatment heterogeneity, it also limits the generalisability of findings to patients treated with other antipsychotics. Thus, while distinctions between first- and second-generation antipsychotics have led to confusion and calls for the classification to be abandoned, individual antipsychotics differ substantially in pharmacological and side-effect profiles (Leucht & Davis, 2011) and may have differential effects on brain structure.

In conclusion, we provide evidence for brain plasticity associated with antipsychotic treatment in schizophrenia. Cortical thickness reductions were unrelated to treatment, while white matter and basal ganglia volume increases were linked to both efficacy and adverse effects. Volume reductions related to illness progression may be more apparent in non-adherent, treatment refractory, chronic samples.
